# Cerebrospinal Fluid Osteopontin and Inflammation-Associated Cytokines in Patients With Anti-*N*-Methyl-D-Aspartate Receptor Encephalitis

**DOI:** 10.3389/fneur.2020.519692

**Published:** 2020-11-05

**Authors:** Cong Zou, Shanshan Pei, Wei Yan, Qingbo Lu, Xiaomei Zhong, Qiong Chen, Suyue Pan, Zhanhang Wang, Honghao Wang, Dong Zheng

**Affiliations:** ^1^Department of Neurology, The Affiliated Brain Hospital of Guangzhou Medical University, Guangzhou, China; ^2^Department of Neurology, Nanfang Hospital, Southern Medical University, Guangzhou, China; ^3^Department of Neurology, The First People's Hospital of Kashgar Prefecture, Kashgar, China; ^4^Department of Neurology, Guangdong 999 Brain Hospital, Guangzhou, China

**Keywords:** osteopontin, anti-*N*-methyl-d-aspartate receptor encephalitis, modified Rankin Scale, cytokine, cerebrospinal fluid

## Abstract

Anti-*N*-methyl-d-aspartate receptor (anti-NMDAR) encephalitis is an autoimmune neurological disorder. Osteopontin (OPN) is a secreted multifunctional phosphorylated glycoprotein that regulates various autoimmune and inflammatory diseases, but its diagnostic and prognostic values in anti-NMDAR encephalitis patients remain elusive. This retrospective study aimed to determine the levels of OPN and related cytokines in cerebrospinal fluid (CSF) of anti-NMDAR encephalitis patients and to assess their influence on disease severity so as to evaluate their efficacy as biomarkers for diagnosis and prognosis. CSF samples from 33 anti-NMDAR encephalitis, 13 viral encephalitis, and 21 controls were collected. All CSF were tested for levels of OPN and inflammation-associated cytokines [interleukin (IL)-6, IL-10, and tumor necrosis factor (TNF)-α] via ELISA. In addition, 15 anti-NMDAR encephalitis patients without follow-up relapse were re-examined for these four parameters 3 months later. The clinical status was evaluated by modified Rankin Scale (mRS) scores. Results showed that the CSF levels of these cytokines were increased in anti-NMDAR encephalitis patients compared to controls but not viral encephalitis patients. Their levels were decreased in remission compared with that in acute stage. Moreover, CSF OPN positively correlated with IL-6, white blood cell (WBC) counts, and C-reactive protein (CRP) levels in anti-NMDAR encephalitis patients. However, no associations were found between OPN or related cytokines and mRS scores in acute stage in anti-NMDAR encephalitis patients. Overall, CSF OPN and related cytokines were increased when anti-NMDAR encephalitis patients are in acute stage and decreased in remission, suggesting the underlying neuro-inflammatory process in this disease. However, they are not qualified with diagnostic or prognostic value.

## Introduction

Anti-*N*-methyl-d-aspartate (anti-NMDA)-receptor encephalitis is newly accepted as an autoimmune neurological disorder mainly affecting young women (1, 2). Typical clinical manifestation of this disease predominantly involves the progressive development of neurological and psychiatric symptoms, such as movement disorder, seizures, speech disorder, consciousness disturbances, autonomic instability, and central hypoventilation (3). Although the production of antibodies to the NMDA receptor is considered to be the main culprit of this disorder, its pathogenesis remains unclear. Patients usually develop from influenza-like symptoms to profound neurological impairments in a short time, and multidisciplinary care becomes essential and urgent (4, 5). It is worth noting that as long as it is treated in time, this disease can be cured with a high probability. However, lack of specific biomarker despite antibodies against NMDAR is a challenge for prompt and accurate diagnosis (6). In addition, anti-NMDAR antibodies have been suggested to have a correlation with outcome or relapses by some articles (7) but not others (8). Cytokines mentioned previously like interleukin (IL)-6, tumor necrosis factor (TNF)-α have been proposed to play important roles in the pathogenesis of this disease (9–11). We also have reported increased inflammatory cytokines in cerebrospinal fluid (CSF) in the acute stage of anti-NMDAR encephalitis (12, 13).

Osteopontin (OPN) is a multifunctional and highly phosphorylated glycoprotein that influences diverse physiological and pathological processes, especially inflammatory and autoimmune response (14–16). It can be secreted by various tissues and cell types and distributed in all body fluids and has been classified as a Th1 cytokine (17, 18). Functions of OPN predominantly presents as regulating cell-mediated immune responses, which involves in various autoimmune and chronic inflammatory diseases. Recently, researchers suggested its pathogenic role in lots of disorders, such as multiple sclerosis, aneurysmal subarachnoid hemorrhage, and glomerulonephritis. Moreover, the upregulated secretion of OPN in plasma or CSF exacerbates most of these diseases (19). Further, OPN neutralization may downregulate the activation of STAT3 signaling pathway (20), which can induce human B and T cells to secrete TNF-α, IL-6, and IL-10 (21). A recent article has found an elevation of CSF OPN concentration in anti-NMDAR encephalitis patients and endowed a predicted role of OPN in the prognosis (22). Here, we aimed to measure CSF levels of OPN and related inflammatory cytokines in anti-NMDAR encephalitis patients so as to investigate any association between these inflammation-associated factors and prognosis and to evaluate the diagnostic and prognostic value of CSF OPN and related cytokines.

In this study, we confirm the elevation of CSF OPN, IL-6, TNF-α, and IL-10 in anti-NMDAR encephalitis patients compared to controls. More importantly, their levels were decreased in the follow-up period compared with that in acute stage. We further explored the associations between CSF OPN, IL-6, TNF-α, and IL-10 and mRS scores in patients with anti-NMDAR encephalitis and investigated an important role of neuroinflammation in the severity of anti-NMDAR encephalitis.

## Method

### Patients and Controls

During 2015 January 1 to 2019 October 30, we enrolled 33 anti-NMDA receptor encephalitis patients and 13 viral encephalitis patients [herpes simplex virus (*n* = 4), mumps virus (*n* = 2), Epstein Barr virus (*n* = 3), and varicella zoster virus (*n* = 4)] and 21 controls with non-inflammatory neurological disease [cervical spondylosis (*n* = 5), migraine (*n* = 5), Alzheimer's disease (*n* = 3), Parkinson's disease (*n* = 3), hypertension (*n* = 3), and normal pressure hydrocephalus (*n* = 2)]. CSF was taken from all the subjects within 3 days for diagnosis and differential diagnosis purpose. First-line treatments were corticosteroids, intravenous immunoglobulin, or plasma exchange, which can be used alone or in combination; second-line immunotherapy was azathioprine here. Fifteen anti-NMDAR encephalitis patients without follow-up relapse were re-examined for the concentrations of CSF OPN, IL-6, IL-10, and TNF-α and reassessed mRS scores 3 months later. All anti-NMDAR encephalitis patients were recruited strictly according to the diagnostic criteria of anti-NMDAR encephalitis (3). Approval has been obtained by the Ethics Committee of the Nanfang Hospital of the Southern Medical University (NFEC-2018-095). Every enrolled participant has signed informed consent. Table 1 shows the detailed clinical information of all participants.

**Table 1:** Demographic and clinical features of patients and controls.

	**Anti-NMDAR**	**Viral**	**Controls**
	**encephalitis**	**encephalitis**	**(*n* = 21)**
	**(*n* = 33)**	**(*n* = 13)**	
Gender (female/male)	19/14	8/5	13/8
Age (years)	34.8 ± 17.6	35.7 ± 15.7	35.2 ± 12.9
**CSF routine**			
CSF WBC (×106/L)^a^	6.0 (2.0,17.3)	4.0 (1.8,6.0)	2.0 (1.2,2.6)
CSF TP (g/L, mean ± SD)	0.4 ± 0.4	0.3 ± 0.6	0.3 ± 0.4
CSF Glu (mmol/L, mean ± SD)	3.7 ± 0.8	3.1 ± 1.2	3.0 ± 0.4
CSF CL (mmol/L, mean ± SD)	120.2 ± 6.7	121.3 ± 9.2	126.3 ± 5.7
**Symptom onset (*****n*****, %)**			
Psychiatric symptoms	25 (75.8)	3 (23.1)	–
Memory deficits	1 (3.0)	1 (7.7)	–
Speech disturbances	2 (6.1)	2 (15.4)	–
Seizures	16 (48.5)	1 (7.7)	–
Movement disorders	7 (21.2)	1 (7.7)	–
Loss of consciousness	15 (45.5)	3 (23.1)	–
Central hypoventilation	6 (18.2)	2 (15.4)	–
**mRS scores**			–
mRS in acute stage (mean ± SD)	4.2 ± 0.9	–	–
3-month follow-up mRS (mean ± SD)	2.4 ± 0.8	–	–
**Treatment (*****n*****, %)**		–	–
Sole first-line treatment	22 (66.7)	–	–
Combined first- and second-line treatment	11 (33.3)	–	–
**Tumor comorbidity (*****n*****, %)**			
Ovarian teratoma	2 (6.1)	0	0
**NMDAR antibody positive in CSF**	33	0	0

Age (years) refers to age at sample collection.

WBC, white blood cell; TP, total protein; Glu, glucose; CL, chlorine; SD, standard deviations; mRS, modified Rankin Scale.

a *Data were presented as medians (IQRs, interquartile ranges)*.

### Preparation of CSF Samples

We obtained CSF samples while patients were in acute stage before treatment. Three months after first- and second-line immune therapy, CSFs of 15 anti-NMDAR encephalitis patients were collected again. All samples were centrifuged immediately and then distributed equally into polypropylene tubes for storage at −80°C until assay.

### Detection of OPN and Proinflammatory Cytokines by ELISA

Sandwich ELISA immunosorbent assay kits were purchased and used to quantify inflammatory cytokines OPN, IL-6, IL-10 (Bender MedSystems GmbH, Vienna, Austria) and TNF-α (Cusabio, Wuhan, China), according to the manufacturers' instructions.

### Statistical Analysis

Data were presented as the mean ± standard deviation (normal distribution) or the medians (IQRs) (non-normal distribution). Kruskal–Wallis test was used for comparisons among the three groups, and three “two-group comparisons” were tested by *post-hoc* Dunn test if needed. Spearman correlation coefficient was used to test correlations. Wilcoxon tests was used to compare OPN concentrations and mRS of patients with anti-NMDAR encephalitis in acute phase and 3 months after therapy. P < 0.05 were considered statistically significant. SPSS 24.0 software (SPSS Inc., Chicago, IL, USA) was used to carry out statistical analysis. All figures were performed using GraphPad Prism 7.0 (GraphPad Software, La Jolla, CA, USA).

## Results

### Clinical Features and Demographics

The clinical manifestations and baseline characteristics of three groups are shown in Table 1. As shown in the table, the mean age of anti-NMDAR encephalitis patients, viral encephalitis, and the control groups was 34.8 ± 17.6, 35.7 ± 15.7, and 35.2 ± 12.9 years, respectively. All individuals were age and sex matched. In patients with anti-NMDAR encephalitis, the mRS score was 4.2 ± 0.9 in the acute stage while 2.4 ± 0.8 in the 3-month follow-up. Psychiatric symptoms accounted for the largest proportion of clinical symptoms in anti-NMDAR encephalitis group. All anti-NMDAR encephalitis patients were CSF NMDAR antibody positive.

### CSF Profiles in Anti-NMDAR Encephalitis, Viral Encephalitis, and Control Patients

**Figure 1:**
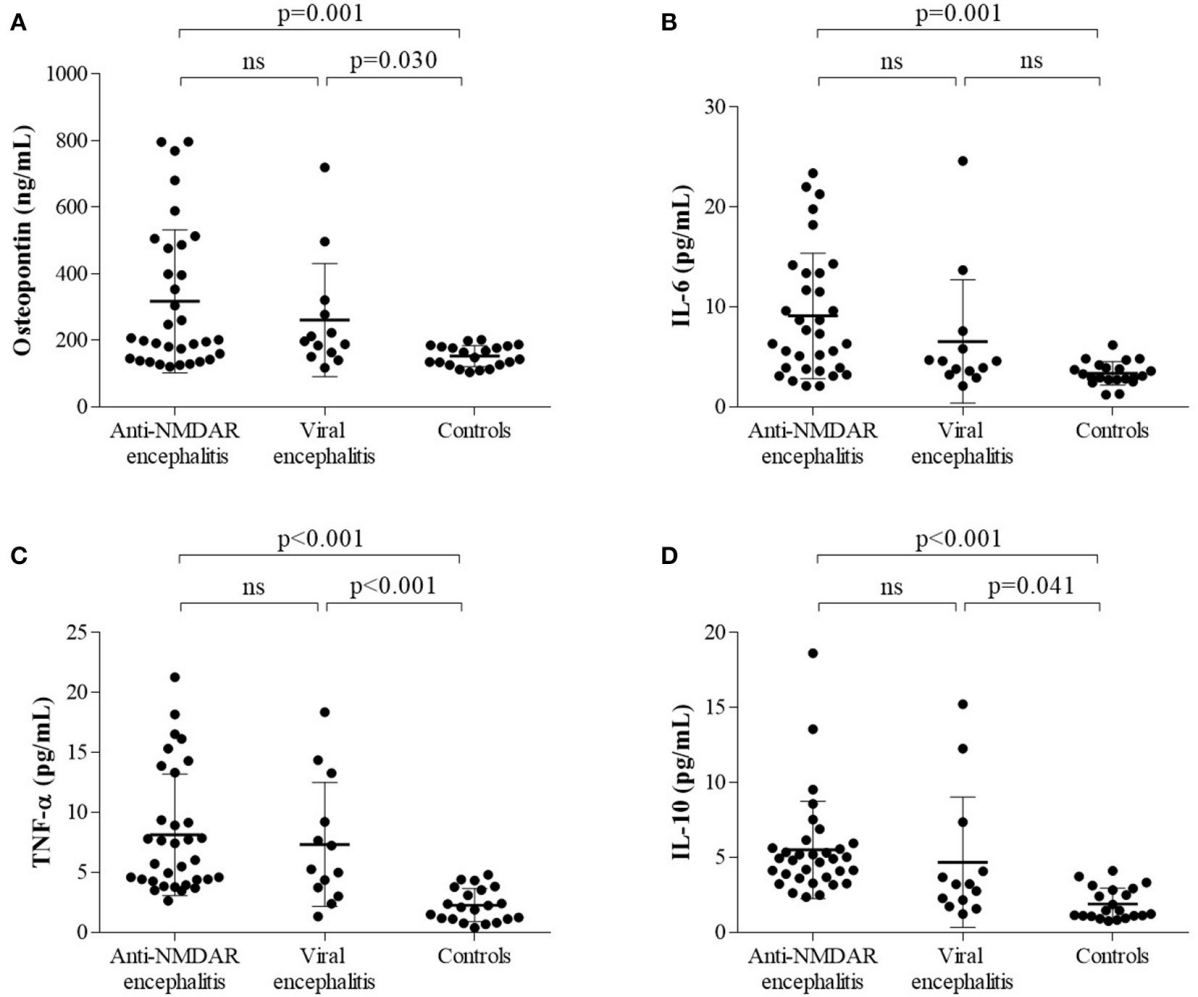
The levels of **(A)** cerebrospinal fluid osteopontin (CSF OPN), **(B)** interleukin (IL)-6, **(C)** tumor necrosis factor alpha (TNF-α), and **(D)** IL-10 in anti-*N*-methyl-d-aspartate receptor (anti-NMDAR) encephalitis patients and two control groups were shown. **(A–D)** Higher CSF levels of OPN, IL-6, TNF-α, and IL-10 were found in anti-NMDAR encephalitis patients than those in controls but not viral encephalitis patients.

**Figure 2:**
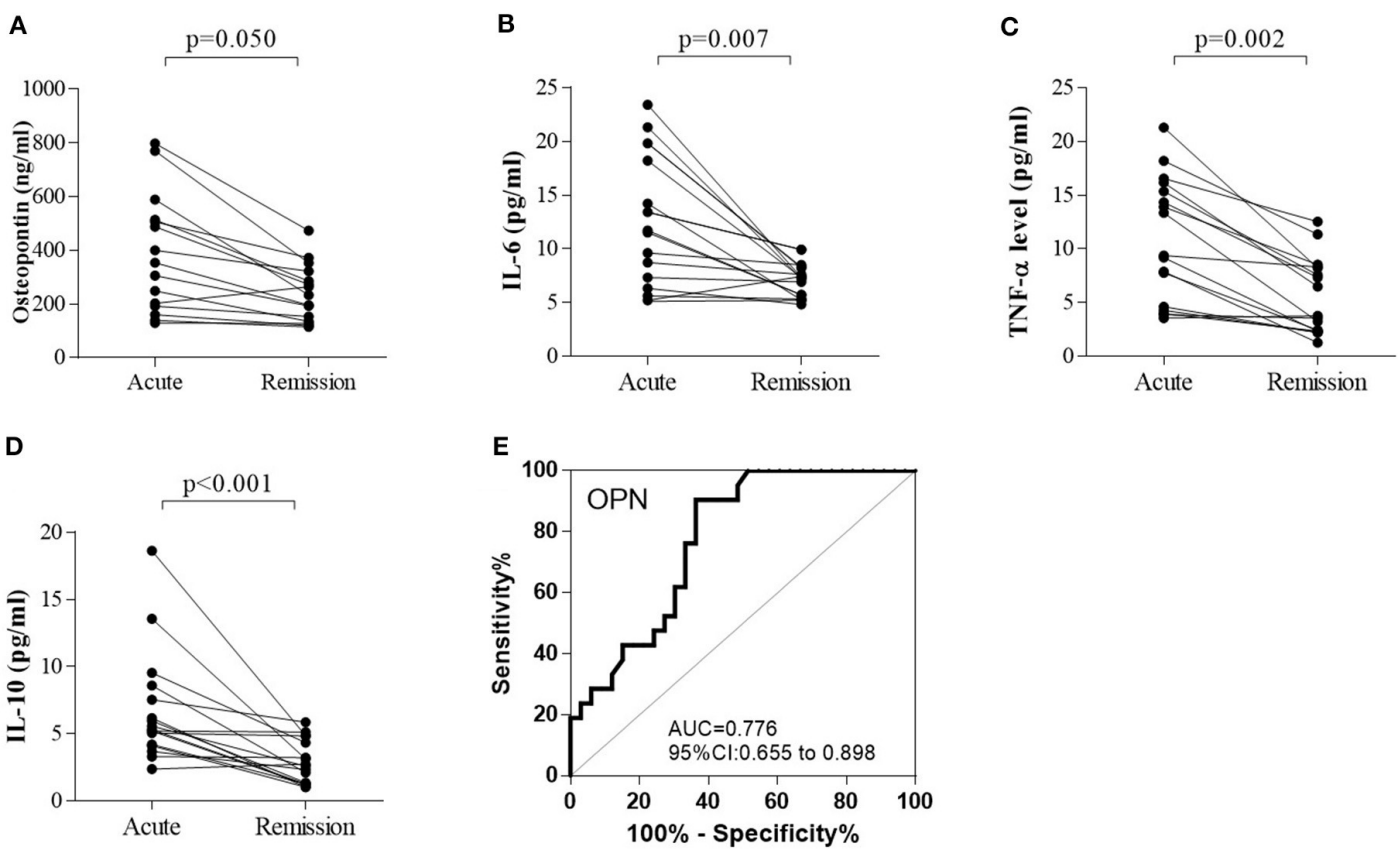
The changes in **(A)** cerebrospinal fluid osteopontin (CSF OPN), **(B)** interleukin (IL)-6, **(C)** tumor necrosis factor alpha (TNF-α), and **(D)** IL-10 in 15 anti-NMDAR encephalitis patients who were examined in both acute and remission stage. **(A–D)** The levels of OPN (*p* = 0.050), IL-6 (*p* = 0.007), TNF-α (*p* = 0.002), and IL-10 (*p* < 0.001) were all significantly decreased in the follow-up period compared with those in acute stage. Receiver operating characteristic (ROC) analyses of **(E)** CSF OPN between patients with anti-NMDAR encephalitis and controls. The area under the receiver operating characteristic (ROC) curve (AUC) of OPN was 0.776.

Both proinflammatory (OPN, IL-6, TNF-α) cytokines and anti-inflammatory cytokine (IL-10) levels were elevated in anti-NMDAR encephalitis patients compared to controls. However, no significant differences were detected between anti-NMDAR encephalitis and viral encephalitis patients. The medians (interquartile ranges) of CSF OPN and other cytokines were significantly higher in the anti-NMDAR encephalitis patients compared to controls in acute stage [OPN 201.5 (143.8, 481.5) vs. 148.4 (126.2, 181.4) ng/ml; *p* = 0.001; Figure 1A; IL-6 7.3 (3.9, 13.4) vs. 3.1 (2.7, 4.1) pg/ml; *p* = 0.001; Figure 1B; TNF-α 6.0 (4.3, 11.3) vs. 2.1 (1.1, 3.7) pg/ml; *p* < 0.001; Figure 1C and IL-10 4.9 (3.6, 5.8) vs. 1.5 (1.1, 2.9) pg/ml; *p* < 0.001; Figure 1D]. For the 15 anti-NMDAR encephalitis patients who were re-examined, the levels of the cytokines were decreased in remission period compared with that in acute stage (Figures 2A–D). We further used receiver operating characteristic (ROC) curves to assess the discriminatory power of OPN to spot anti-NMDAR encephalitis from controls. The areas under the curve (AUC) of OPN was 0.777 (Figure 2E).

### Correlations Between CSF OPN and Proinflammatory Cytokines and mRS in Different Groups and the Follow-Up CSF Samples

**Figure 3:**
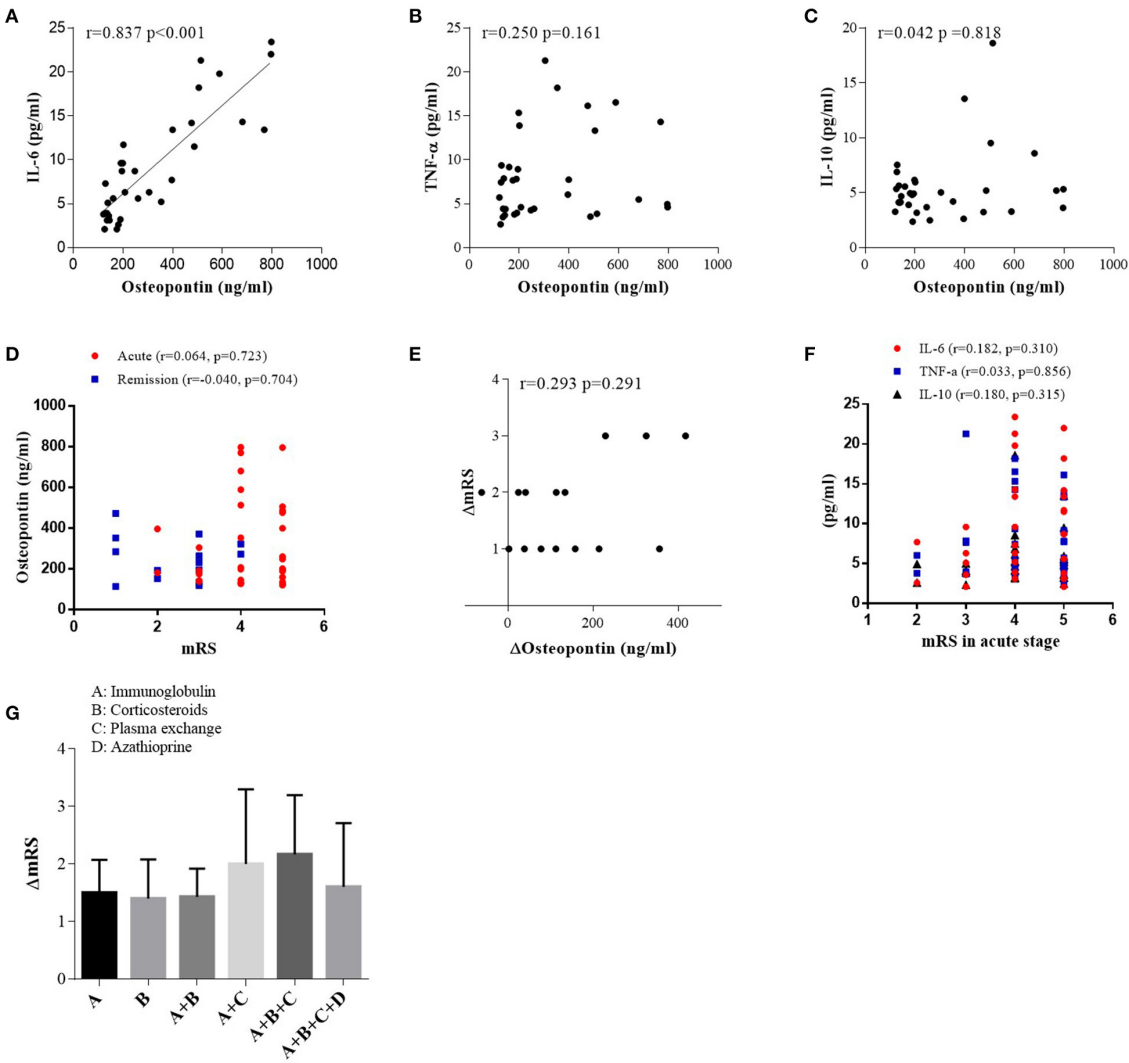
Correlations between cerebrospinal fluid osteopontin (CSF OPN) and **(A)** interleukin (IL)-6, **(B)** tumor necrosis factor alpha (TNF-α), **(C)** IL-10, **(D)** max mRS scores in acute stage, and **(D)** CSF OPN and mRS scores in remission of anti-*N*-methyl-d-aspartate receptor (anti-NMDAR) encephalitis patients. Correlation was found between CSF OPN and IL-6 in acute stage (*r* = 0.837, *p* < 0.001). **(E)** Association between the reduction in mRS scores and the decrease in CSF OPN [ΔmRS = mRS (acute stage) – mRS (follow up), ΔOP *n* = OPN (acute stage) – OPN (follow up)]. No association was found between ΔOPN and ΔmRS in patients with anti-NMDAR encephalitis (*r* = 0.293, *p* = 0.291). **(F)** Correlations between CSF IL-6, TNF-α, IL-10, and mRS scores in acute stage. No association was found between mRS scores and CSF IL-6 (*r* = 0.182, *p* = 0.310), TNF-α (*r* = 0.033, *p* = 0.856), or IL-10 (*r* = 0.180, *p* = 0.315) in acute stage. **(G)** The influence of acute therapy on changes of mRS scores.

In patients with anti-NMDAR encephalitis, we discovered a positive correlation between OPN and IL-6 (*r* = 0.837, *p* < 0.001; Figure 3A) in acute stage. However, there was no correlation between OPN and TNF-α or IL-10 (Figures 3B,C). In patients with viral encephalitis and controls, we only found significant correlations between OPN and TNF-α (*r* = 0.632, *p* = 0.021) in the viral encephalitis patients and IL-10 and TNF-α in the controls (*r* = 0.617, *p* = 0.019) (data shown in Supplementary Materials). However, in patients with anti-NMDAR encephalitis, no correlations were found between both CSF OPN or related cytokines concentrations and mRS scores in acute stage (Figures 3D,F), and CSF OPN concentrations were also not associated with mRS scores in remission (Figure 3D). Similarly, the decrease in CSF OPN levels had no relationship with the reduction in mRS scores (*r* = 0.293, *p* = 0.291) (Figure 3E). Between the changes in cytokines IL-6, IL-10, and TNF-α and the changes in mRS score in anti-NMDAR encephalitis group, only ΔIL-10 and ΔmRS were significantly correlated (data shown in Supplementary Materials). We further calculated the influence of acute therapy on changes in mRS scores, but no significant difference was found in effect among different treatment groups (Figure 3G).

### Clinical Features Related to Elevated CSF OPN Concentrations in Anti-NMDAR Encephalitis Patients

Correlations between CSF levels of OPN and clinical data are shown in Table 2. We discovered that both the CSF OPN concentrations were associated with CSF white blood cell (WBC) count (OPN: *r* = 0.590, *p* = 0.004) and CSF C-reactive protein (CRP) concentrations (OPN: *r* = 0.378, *p* = 0.030). However, no significant associations were found between the OPN levels and age, gender, total protein (TP), glucose, chlorine, or clinical characteristics in the acute phase shown in Table 1.

**Table 2:** Correlations between cerebrospinal fluid (CSF levels of osteopontin (OPN) and clinical data.

	***r***	***p***	**95% CI**
**OPN**			
CSF WBC count	0.590	0.004	0.230–0.812
CSF CRP	0.378	0.030	0.029–0.645

## Discussion

Until now, suitable biomarkers for anti-NMDAR encephalitis are still lacking. Although anti-NMDAR antibodies have been suggested to have a correlation with outcome or relapses by some articles (7), others were in disagreement with this view (8). Inflammation has been known to be important in the development and relapse stage of anti-NMDAR encephalitis (23), and anti-NMDAR encephalitis is a central nervous system (CNS) disorders, so we tried to find out the role of CSF inflammatory cytokines OPN, IL-6, TNF-α, and IL-10 in anti-NMDAR encephalitis.

In this study, we detected a significant increase in CSF OPN, IL-6, TNF-α, and IL-10 in anti-NMDAR encephalitis patients in acute stage compared to controls, and both the levels of CSF OPN and its relevant cytokines decrease as the disease develops into the remission stage. We discovered that both the CSF OPN concentrations were associated with CSF WBC count and CSF CRP concentrations. There was also a positive correlation between CSF OPN levels and CSF IL-6 levels in anti-NMDAR encephalitis patients in acute stage. However, no association was found between the CSF OPN or related cytokines concentrations and mRS scores in acute stage of anti-NMDAR encephalitis patients; this may be caused by earlier treatment and insufficient sample size. Altogether, our results suggested that CSF OPN and related cytokines may participate in the underlying inflammatory process of anti-NMDAR encephalitis but may not affect the disease severity. Moreover, these detections revealed that OPN and its relevant cytokines were remarkably activated in anti-NMDAR encephalitis and gradually decreased with clinical improvement, which confirm the pathogenic role of OPN and its relevant cytokines in anti-NMDAR encephalitis. Interestingly, the cytokines levels are not significantly different between the anti-NMDAR encephalitis group and viral encephalitis group, suggesting that the two groups share some common pathogenesis, which seems that their utility are limited in the field of early differential diagnosis of anti-NMDAR encephalitis from controls. Moreover, the levels of CSF OPN levels were correlated with CSF WBC count and CSF CRP levels. ROC analysis showed discriminatory power of OPN and related cytokines for spotting anti-NMDAR encephalitis from controls. Therefore, CSF OPN and related cytokines have the potential to change the inflammatory milieu, implying that further study on associations between CSF OPN, related cytokines, and other clinical elements like MRI data is worthy.

Similarly, a recent article also figured out an increased CSF OPN in patients with anti-NMDAR encephalitis and speculated a predicted role of OPN in the prognosis of anti-NMDAR encephalitis patients (21). In this study, we confirmed the elevation of CSF OPN in anti-NMDAR encephalitis patients, but OPN is not qualified with diagnostic or prognostic value in some cases, which may be affected by early treatment that patients received before CSF extraction. Here, we further explored the CSF levels of related inflammatory cytokines IL-6, TNF-α, and IL-10 in these patients.

Although the role of B cells in anti-NMDAR encephalitis has already been demonstrated (24, 25), the mechanism of B cell activation remains unclear. OPN is a multifunctional proinflammatory cytokine that can be secreted from many cells, including activated macrophages and T lymphocytes. Some authors proposed that OPN can induce B lymphocyte proliferation and immunoglobulin production. Recently, the role of cytokines from T cells in the pathogenesis of anti-NMDAR encephalitis has been proposed more frequently by researchers. As a novel Th1 cytokine, OPN influences cell-mediated immunity by contributing to the Th1 and Th17 response (10, 26). Altogether, the above suggests a pathogenic role of OPN in anti-NMDAR encephalitis.

OPN is classified as a Th1 cytokine that regulates cell-mediated immune responses. Inflammatory signals can upregulate the production of OPN, especially in CNS. OPN exists in sites of inflammation and is secreted by active leukocytes and macrophages (26). Moreover, Shinohara et al. reported that T-bet, a transcription factor that controls Th1 responses, regulates the expression of OPN (27). Thus, OPN act as an indicator of the inflammatory response. The increase in OPN have been demonstrated in many inflammatory autoimmune diseases, such as multiple sclerosis, glomerulonephritis, Crohn's disease, and systemic lupus erythematosus (SLE) (19, 28–31), which suggests that OPN plays a significant role in the pathogenesis of these inflammatory autoimmune disease. In fact, many researches have demonstrated that the OPN gene knockout animal could suffer less from the inflammation of autoimmune and non-immune diseases (19, 32–37).

Cytokines especially IL-6 has been suggested to play a pathogenic role in anti-NMDAR encephalitis (12, 38). Moreover, IL-6, which could regulate Th17/Treg (regulatory T cells) balance (39), is also increased in CNS autoimmune diseases, such as NMO (40). OPN is known to be induced in macrophages by several inflammatory cytokines, including IL-6, and some studies also indicated that OPN promotes secretion of IL-6. Scatena, M. et al. showed that by modulating phosphorylation of kinases [nuclear factor kappa B (NF-κB) inducing kinase (NIK), inhibitory-κB kinase β (IKKβ)], NF-κB is subsequently activated and then enhanced the expression of a lot of inflammatory mediators such as IL-β, the upstream of IL-6 (17). OPN may regulate immune responses and promote secretion of IL-6 through activation of the NF-κB pathway. Although OPN is known to be induced in macrophages by TNF-α, the influence of OPN on TNF-α remains unknown. These may be the reasons for the positive correlation between OPN and IL-6 but not with TNF-α. Our study also found the increase in IL-6 in anti-NMDAR encephalitis patients, and there were positive correlations between the CSF OPN and IL-6, which suggests that OPN/IL-6 coactivation in anti-NMDAR encephalitis might be the key factor for disease pathogenesis.

We also found that anti-inflammatory cytokine IL-10 was higher in anti-NMDAR encephalitis in comparison to the controls. Moreover, our previous studies also revealed an elevation of IL-10 in patients with anti-NMDAR encephalitis (13, 41). However, Zohar et al. suggested that OPN suppressed the secretion of macrophage-directed IL-10 through interaction with CD44 on macrophages (17). Thus, the phenomenon that both OPN and IL-10 were increased in anti-NMDAR encephalitis is contradictory. Considering the widely accepted anti-inflammatory and immunosuppressive role of IL-10 (42), the increase in CSF IL-10 may attribute to some unknown negative feedback protective regulation in anti-NMDAR encephalitis. In this study, OPN may not be qualified to act as an indicator for anti-NMDAR encephalitis to tell itself from viral encephalitis. The insignificant difference between anti-NMDAR encephalitis and viral encephalitis in our study may be due to the small sample size.

Our data support the idea that CSF OPN may have pleiotropic functions in the pathogenesis of inflammatory disease; more importantly, CSF OPN, IL-6, TNF-α, and IL-10 maybe involved in the underlying neuro-inflammatory process of anti-NMDAR encephalitis. At the same time, our findings regarding OPN, IL-6, IL-10, and TNF-α raise the question of possible involvement of T cells in anti-NMDAR encephalitis. However, certain limitations should also be emphasized in the present study. First, patients' clinical data were incomplete based on its retrospective background, and the number of patients was relatively small. Second, OPN is not routinely available in most laboratories at present, making it hard to fulfill its utility clinically. Third, this study did not detect paired serum samples due to the improper storage of samples. Considering that patients with anti-NMDAR encephalitis in remission do not usually undergo seriated lumbar punctures unless the recovery is unsatisfactory, further study should focus on finding serum markers that correlates with disease activity.

## Conclusion

In this study, we revealed that the CSF levels of OPN, IL-6, IL-10, and TNF-α were obviously higher in anti-NMDAR encephalitis patients in acute stage compare to those in remission. Moreover, CSF levels of the above cytokines were higher in anti-NMDAR encephalitis patients than in controls but not viral encephalitis patients. However, CSF OPN and the related cytokines were not associated with disease severity of anti-NMDAR encephalitis, which did not support that OPN or related cytokines can predict the severity of the disease, but these results might be affected by earlier treatment and insufficient sample size. In addition, we found a positive correlation between CSF OPN and the CSF IL-6, WBC counts, and CRP levels. In conclusion, CSF OPN and related cytokines levels were increased in patients with anti-NMDAR encephalitis, reflecting the underlying neuro-inflammatory processes.

## Data Availability Statement

All datasets generated for this study are included in the article/Supplementary Material.

## Ethics Statement

The studies involving human participants were reviewed and approved by Ethics Committee of the Nanfang Hospital of Southern Medical University. The patients/participants provided their written informed consent to participate in this study. Written informed consent was obtained from the individual(s) for the publication of any potentially identifiable images or data included in this article.

## Author Contributions

DZ, ZW, and HW designed the experiments and revised the article. CZ, SPe, and WY collected the samples and clinical data. SPe and CZ performed the experiments and wrote the manuscript. QL, XZ, QC, and SPa analyzed the data and searched the literature. All authors contributed to the revision of the manuscript and approved the submitted version.

## Conflict of Interest

The authors declare that the research was conducted in the absence of any commercial or financial relationships that could be construed as a potential conflict of interest.

## References

[1] Titulaer MJ McCracken L Gabilondo I Armangue T Glaser C Iizuka T. Treatment and prognostic factors for long-term outcome in patients with anti-NMDA receptor encephalitis: an observational cohort study. Lancet Neurol. (2013) 12:157–65. 10.1016/S1474-4422(12)70310-1 23290630 PMC3563251

[2] Chen X Li JM Liu F Wang Q Zhou D Lai X. Anti-*N*-methyl-D-aspartate receptor encephalitis: a common cause of encephalitis in the intensive care unit. Neurol Sci. (2016) 37:1993–8. 10.1007/s10072-016-2702-y 27620725

[3] Graus F Titulaer MJ Balu R Benseler S Bien CG Cellucci T. A clinical approach to diagnosis of autoimmune encephalitis. Lancet Neurol. (2016) 15:391–404. 10.1016/S1474-4422(15)00401-9 26906964 PMC5066574

[4] Vincent A Bien CG. Anti-NMDA-receptor encephalitis: a cause of psychiatric, seizure, and movement disorders in young adults. Lancet Neurol. (2008) 7:1074–5. 10.1016/S1474-4422(08)70225-4 18851929

[5] Endres D Rauer S Kern W Venhoff N Maier SJ Runge K. Psychiatric presentation of anti-NMDA receptor encephalitis. Front Neurol. (2019) 10:1086. 10.3389/fneur.2019.01086 31749755 PMC6848057

[6] Liu CY Zhu J Zheng XY Ma C Wang X. Anti-*N*-methyl-D-aspartate receptor encephalitis: a severe, potentially reversible autoimmune encephalitis. Mediat Inflamm. (2017) 2017:6361479. 10.1155/2017/6361479 28698711 PMC5494059

[7] Gresa-Arribas N Titulaer MJ Torrents A Aguilar E McCracken L Leypoldt F. Antibody titres at diagnosis and during follow-up of anti-NMDA receptor encephalitis: a retrospective study. Lancet Neurol. (2014) 13:167–77. 10.1016/S1474-4422(13)70282-5 24360484 PMC4006368

[8] Hansen HC Klingbeil C Dalmau J Li W Weissbrich B Wandinger KP. Persistent intrathecal antibody synthesis 15 years after recovering from anti-*N*-methyl-D-aspartate receptor encephalitis. JAMA Neurol. (2013) 70:117–9. 10.1001/jamaneurol.2013.585 23318518 PMC3707142

[9] Tuzun E Zhou L Baehring JM Bannykh S Rosenfeld MR Dalmau J. Evidence for antibody-mediated pathogenesis in anti-NMDAR encephalitis associated with ovarian teratoma. Acta Neuropathol. (2009) 118:737–43. 10.1007/s00401-009-0582-4 19680671 PMC2888642

[10] Liba Z Kayserova J Elisak M Marusic P Nohejlova H Hanzalova J. Anti-*N*-methyl-D-aspartate receptor encephalitis: the clinical course in light of the chemokine and cytokine levels in cerebrospinal fluid. J Neuroinflammation. (2016) 13:55. 10.1186/s12974-016-0507-9 26941012 PMC4776396

[11] Wang X Ma C Liu CY Li GJ Zhao D Han DF. Neuronal NMDAR Currents of the hippocampus and learning performance in autoimmune anti-NMDAR encephalitis and involvement of TNF-alpha and IL-6. Front Neurol. (2019) 10:684. 10.3389/fneur.2019.00684 31297084 PMC6607466

[12] Ai P Zhang X Xie Z Liu G Liu X Pan S. The HMGB1 is increased in CSF of patients with an anti-NMDAR encephalitis. Acta Neurol Scand. (2018) 137:277–82. 10.1111/ane.12850 29023630

[13] Chen J Ding Y Zheng D Wang Z Pan S Ji T. Elevation of YKL-40 in the CSF of anti-NMDAR encephalitis patients is associated with poor prognosis. Front Neurol. (2018) 9:727. 10.3389/fneur.2018.00727 30369903 PMC6194180

[14] Denhardt DT Giachelli CM Rittling SR. Role of osteopontin in cellular signaling and toxicant injury. Annu Rev Pharmacol Toxicol. (2001) 41:723–49. 10.1146/annurev.pharmtox.41.1.723 11264474

[15] Scatena M Liaw L Giachelli CM. Osteopontin: a multifunctional molecule regulating chronic inflammation and vascular disease. Arterioscler Thromb Vasc Biol. (2007) 27:2302–9. 10.1161/ATVBAHA.107.144824 17717292

[16] Rittling SR Singh R. Osteopontin in immune-mediated diseases. J Dent Res. (2015) 94:1638–45. 10.1177/0022034515605270 26341976 PMC4681477

[17] Zohar R Suzuki N Suzuki K Arora P Glogauer M McCulloch CA. Intracellular osteopontin is an integral component of the CD44-ERM complex involved in cell migration. J Cell Physiol. (2000) 184:118–30. 10.1002/(SICI)1097-4652(200007)184:1<118::AID-JCP13>3.0.CO;2-Y 10825241

[18] Borges K Gearing M Rittling S Sorensen ES Kotloski R Denhardt DT. Characterization of osteopontin expression and function after status epilepticus. Epilepsia. (2008) 49:1675–85. 10.1111/j.1528-1167.2008.01613.x 18522644 PMC4090704

[19] Lund SA Giachelli CM Scatena M. The role of osteopontin in inflammatory processes. J Cell Commun Signal. (2009) 3:311–22. 10.1007/s12079-009-0068-0 19798593 PMC2778587

[20] Sangaletti S Tripodo C Sandri S Torselli I Vitali C Ratti C. Osteopontin shapes immunosuppression in the metastatic niche. Cancer Res. (2014) 74:4706–19. 10.1158/0008-5472.CAN-13-3334 25035397

[21] Agrawal S Gollapudi S Su H Gupta S. Leptin activates human B cells to secrete TNF-alpha, IL-6, and IL-10 via JAK2/STAT3 and p38MAPK/ERK1/2 signaling pathway. J Clin Immunol. (2011) 31:472–8. 10.1007/s10875-010-9507-1 21243519 PMC3132280

[22] Zhao J Wang C Zhang Y Sun R Wang H Li G. Elevated CHI3L1 and OPN levels in patients with anti-*N*-methyl-d-aspartate receptor encephalitis. J Neuroimmunol. (2019) 334:577005. 10.1016/j.jneuroim.2019.577005 31310926

[23] Liu B Ai P Zheng D Jiang Y Liu X Pan S. Cerebrospinal fluid pentraxin 3 and CD40 ligand in anti-*N*-menthyl-d-aspartate receptor encephalitis. J Neuroimmunol. (2018) 315:40–4. 10.1016/j.jneuroim.2017.11.016 29306404

[24] Hachiya Y Uruha A Kasai-Yoshida E Shimoda K Satoh-Shirai I Kumada S. Rituximab ameliorates anti-*N*-methyl-D-aspartate receptor encephalitis by removal of short-lived plasmablasts. J Neuroimmunol. (2013) 265:128–30. 10.1016/j.jneuroim.2013.09.017 24183642

[25] Dalmau J Lancaster E Martinez-Hernandez E Rosenfeld MR Balice-Gordon R. Clinical experience and laboratory investigations in patients with anti-NMDAR encephalitis. Lancet Neurol. (2011) 10:63–74. 10.1016/S1474-4422(10)70253-2 21163445 PMC3158385

[26] Wang KX Denhardt DT. Osteopontin: role in immune regulation and stress responses. Cytokine Growth Factor Rev. (2008) 19:333–45. 10.1016/j.cytogfr.2008.08.001 18952487

[27] Shinohara ML Jansson M Hwang ES Werneck MB Glimcher LH Cantor H. T-bet-dependent expression of osteopontin contributes to T cell polarization. Proc Natl Acad Sci USA. (2005) 102:17101–6. 10.1073/pnas.0508666102 16286640 PMC1288014

[28] Mishima R Takeshima F Sawai T Ohba K Ohnita K Isomoto H. High plasma osteopontin levels in patients with inflammatory bowel disease. J Clin Gastroenterol. (2007) 41:167–72. 10.1097/MCG.0b013e31802d6268 17245215

[29] Agnholt J Kelsen J Schack L Hvas CL Dahlerup JF Sorensen ES. Osteopontin, a protein with cytokine-like properties, is associated with inflammation in Crohn's disease. Scand J Immunol. (2007) 65:453–60. 10.1111/j.1365-3083.2007.01908.x 17444956

[30] Comabella M Pericot I Goertsches R Nos C Castillo M Blas Navarro J. Plasma osteopontin levels in multiple sclerosis. J Neuroimmunol. (2005) 158:231–9. 10.1016/j.jneuroim.2004.09.004 15589058

[31] Uaesoontrachoon K Wasgewatte Wijesinghe DK Mackie EJ Pagel CN. Osteopontin deficiency delays inflammatory infiltration and the onset of muscle regeneration in a mouse model of muscle injury. Dis Models Mech. (2013) 6:197–205. 10.1242/dmm.009993 22917925 PMC3529351

[32] Jansson M Panoutsakopoulou V Baker J Klein L Cantor H. Cutting edge: attenuated experimental autoimmune encephalomyelitis in eta-1/osteopontin-deficient mice. J Immunol. (2002) 168:2096–9. 10.4049/jimmunol.168.5.2096 11859094

[33] Yumoto K Ishijima M Rittling SR Tsuji K Tsuchiya Y Kon S. Osteopontin deficiency protects joints against destruction in anti-type II collagen antibody-induced arthritis in mice. Proc Natl Acad Sci USA. (2002) 99:4556–61. 10.1073/pnas.052523599 11930008 PMC123686

[34] Frenzel DF Borkner L Scheurmann J Singh K Scharffetter-Kochanek K Weiss JM. Osteopontin deficiency affects imiquimod-induced psoriasis-like murine skin inflammation and lymphocyte distribution in skin, draining lymph nodes and spleen. Exp Dermatol. (2015) 24:305–7. 10.1111/exd.12649 25655893

[35] Oz HS Zhong J de Villiers WJ. Osteopontin ablation attenuates progression of colitis in TNBS model. Dig Dis Sci. (2012) 57:1554–61. 10.1007/s10620-011-2009-z 22173746

[36] Hashimoto M Sun D Rittling SR Denhardt DT Young W. Osteopontin-deficient mice exhibit less inflammation, greater tissue damage, and impaired locomotor recovery from spinal cord injury compared with wild-type controls. J Neurosci. (2007) 27:3603–11. 10.1523/JNEUROSCI.4805-06.2007 17392476 PMC6672107

[37] Zeng C Chen L Chen B Cai Y Li P Yan L. Th17 cells were recruited and accumulated in the cerebrospinal fluid and correlated with the poor prognosis of anti-NMDAR encephalitis. Acta Biochim Biophys Sin. (2018) 50:1266–73. 10.1093/abbs/gmy137 30418472

[38] Kimura A Kishimoto T. IL-6: regulator of Treg/Th17 balance. Eur J Immunol. (2010) 40:1830–5. 10.1002/eji.201040391 20583029

[39] Uzawa A Mori M Arai K Sato Y Hayakawa S Masuda S. Cytokine and chemokine profiles in neuromyelitis optica: significance of interleukin-6. Mult Scler. (2010) 16:1443–52. 10.1177/1352458510379247 20739337

[40] Peng Y Zheng D Zhang X Pan S Ji T Zhang J. Cell-free mitochondrial DNA in the CSF: a potential prognostic biomarker of anti-NMDAR encephalitis. Front Immunol. (2019) 10:103. 10.3389/fimmu.2019.00103 30792710 PMC6375341

[41] Ouyang W O'Garra A. IL-10 Family cytokines IL-10 and IL-22: from basic science to clinical translation. Immunity. (2019) 50:871–91. 10.1016/j.immuni.2019.03.020 30995504

[42] Masutani K Akahoshi M Tsuruya K Tokumoto M Ninomiya T Kohsaka T. Predominance of Th1 immune response in diffuse proliferative lupus nephritis. Arthritis Rheum. (2001) 44:2097–106. 10.1002/1529-0131(200109)44:9<2097::AID-ART360>3.0.CO;2-6 11592372

