# Supplementary material for: Cerebrospinal Fluid Osteopontin and Inflammation-Associated Cytokines in Patients With Anti-N-Methyl-D-Aspartate Receptor Encephalitis
Source: Front Neurol. 2020 Nov 5;11:519692. doi: 10.3389/fneur.2020.519692 (PMC7676223; doi:10.3389/fneur.2020.519692)
Supplement: Supplementary file 1 [file Table_1.DOCX]

**Supplementary Table 1: Correlations between inflammatory cytokines and mRS in three groups (*r*).**

|  | anti-NMDAR encephalitis  OPN IL-6 TNF-α IL-10 ΔIL-6 ΔTNF-α ΔIL-10 | Viral encephalitis  OPN IL-6 TNF-α IL-10 | Controls  OPN IL-6 TNF-α IL-10 |
| --- | --- | --- | --- |
| OPN | 1.000 0.837***0.250 0.042 NA NA NA | 1.000 0.509 0.632* 0.324 | 1.000 -0.180 -0.184 -0.156 |
| IL-6 | 0.837*** 1.000 0.291 0.180 NA NA NA | 0.509 1.000 0.234 0.327 | -0.180 1.000 0.147 0.128 |
| TNF-α  IL-10  mRS  ΔmRS | 0.250 0.291 1.000 0.122 NA NA NA  0.042 0.180 0.122 1.000 NA NA NA  0.064 0.182 0.033 0.180 NA NA NA  NA NA NA NA 0.371 0.189 0.625* | 0.632* 0.234 1.000 -0.126  0.324 0.327 -0.126 1.000  NA NA NA NA  NA NA NA NA | -0.184 0.147 1.000 0.617*  -0.156 0.128 0.617* 1.000  NA NA NA NA  NA NA NA NA |

Δ= cytokines or mRS (acute stage)- cytokines or mRS (follow‐up). * P<0.05, ** P<0.01, ***P<0.001.
